# Probiotics as Emerging Immunoredox Modulators: Insights into the Microbiota–Mitochondria Axis in Oxidative and Autoimmune-Mediated Disorders

**DOI:** 10.3390/antiox15101288

**Published:** 2026-10-03

**Authors:** Max Denisson Maurício Viana, Diego Domingues Pereira, Sthefane Silva Santos, Maria Laura Camilo Calderari, Vinícius Eduardo Fernandes da Silva, Marvin Paulo Lins, Luiz Henrique Agra Cavalcante-Silva

**Affiliations:** 1 School of Pharmacy, Federal University of Bahia, Salvador 40170-290, BA, Brazil; diegodomingues@ufba.br (D.D.P.); sthefaness@ufba.br (S.S.S.); 2 Laboratory of Immunology, Medical School, Federal University of Mato Grosso, Cuiabá 78060-900, MT, Brazil; maria.calderari@sou.ufmt.br (M.L.C.C.); marvin.lins@ufmt.br (M.P.L.); 3 Postgraduate Program in Pharmaceutical Sciences, Institute of Pharmaceutical Sciences, Federal University of Alagoas, Maceió 57072-900, AL, Brazil; vinicius.silva@icf.ufal.br (V.E.F.d.S.); luiz.agra@arapiraca.ufal.br (L.H.A.C.-S.)

**Keywords:** oxidative stress, inflammation, antioxidant signaling, immunoredox, immunometabolism, microbiota, gut–mitochondria axis, gut–immune axis, probiotics

## Abstract

Research to date has predominantly focused on probiotic-induced microbiota modulation, anti-inflammatory effects, and generalized antioxidant activity. However, evidence regarding the role of probiotics in regulating the microbiota–mitochondria axis and its impact on immunoredox regulation remains fragmented and poorly characterized. This review discusses probiotics as immunoredox therapeutic modulators, rather than solely microbiota regulators, offering a conceptual framework with translational relevance. This manuscript explores how probiotics influence ROS/RNS production, Nrf2/NF-κB signaling, mitochondrial redox homeostasis, inflammatory immunometabolism, and antioxidant defenses in immune cells. Additionally, the review integrates emerging evidence linking these mechanisms to oxidative stress and autoimmunity. By focusing on mechanistic and translational aspects, this review aims to provide new insights into probiotic-based strategies targeting oxidative stress and immune dysfunction.

## 1. Introduction

Oxidative stress and immune dysregulation are interconnected hallmarks of numerous chronic disorders, including autoimmune and neurodegenerative diseases [1]. Although oxidative stress has traditionally been viewed as resulting from excessive production of reactive oxygen species (ROS) that overwhelms antioxidant defenses, advances in redox biology have revealed that ROS also serve as essential signaling mediators, that regulate immune cell activation, differentiation, and inflammatory responses [2]. Under physiological conditions, tightly controlled redox signaling supports immune homeostasis, whereas its disruption promotes inflammatory activation, loss of immune tolerance, and tissue injury [3].

These observations highlight the immunoredox interface, through which redox signaling links cellular metabolism to immune regulation [4]. Within this framework, mitochondria have emerged as central regulators of immune function. Beyond their classical bioenergetic functions, they generate mitochondrial ROS (mtROS) and integrate metabolic and inflammatory signaling [5]. Accordingly, disturbances in mitochondrial function and redox homeostasis are now recognized as key mechanistic contributors to the initiation and progression of immune-mediated and neurodegenerative diseases [6].

Growing evidence suggests that the gut microbiota (GM) is a key regulator of immune homeostasis through mechanisms involving mitochondrial function and redox signaling [7,8]. Among GM-derived bioactive metabolites, short-chain fatty acids (SCFAs) play important roles in immune cell differentiation, inflammatory responses, mitochondrial metabolism, tissue homeostasis, and the maintenance of microbial ecosystem balance. These findings have led to the concept of the microbiota–mitochondria axis (MMA), a bidirectional network in which microbial metabolites and signals shape mitochondrial function, while mitochondrial metabolism and signaling, in turn, influence the gut microbiota and host immune responses [9]. In this context, probiotics, defined as live microorganisms that confer a health benefit on the host when administered in adequate amounts, have emerged as promising immunoredox modulators [10]. By modulating GM composition and metabolite production, probiotics may help restore redox and immune homeostasis [11,12].

Despite growing evidence supporting the involvement of the MMA in immune and redox regulation, the mechanisms through which probiotic interventions may influence this bidirectional network remain poorly characterized across different disease contexts. Moreover, probiotics have rarely been examined from an immunoredox perspective that integrates microbial, mitochondrial, and immune signaling. This review uses the MMA as an interpretive framework to integrate and critically discuss evidence linking probiotic interventions to microbiota-derived metabolic signaling, mitochondrial function, and immunoredox regulation. This perspective brings together findings that are often considered separately, allowing the potential interplay among these processes to be examined across oxidative stress and autoimmunity, while distinguishing established mechanisms from associations that require experimental validation.

## 2. Immunoredox Signaling and Immune Dysfunction

### 2.1. Physiological Versus Pathological ROS Production

The distinction between physiological and pathological ROS production is not merely quantitative. Physiological redox signaling involves transient, spatially confined, and enzymatically regulated ROS generation, particularly hydrogen peroxide (H_2_O_2_), which diffuses across membranes and reversibly oxidizes redox-sensitive protein residues [13]. By modifying cysteine thiols in kinases, phosphatases, transcription factors, and adaptor proteins, H_2_O_2_ regulates signaling pathways involved in immune activation and adaptation [14]. This controlled process, termed oxidative eustress, contrasts with oxidative distress, in which sustained or excessive ROS production overwhelms antioxidant defenses and damages lipids, proteins, nucleic acids, and organelles [15].

ROS arise from several enzymatic sources, including the mitochondrial electron transport chain, NADPH oxidases (NOX), xanthine oxidoreductase, peroxisomal oxidases, and endoplasmic reticulum oxidoreductases, with NOX enzymes and mitochondria being particularly important in immune cells [16]. NOX2 supports the phagocyte oxidative burst, whereas mtROS regulates lymphocyte activation, macrophage polarization, and inflammasome signaling [17,18]. Under homeostatic conditions, these oxidants are controlled by glutathione, thioredoxin, peroxiredoxins, catalase, superoxide dismutases (SODs), and nuclear factor erythroid 2-related factor 2 (Nrf2)-dependent pathways [3].

Mitochondrial dysfunction, impaired mitophagy, chronic inflammation, and metabolic stress can disrupt this balance and promote pathological ROS accumulation [19]. Sustained exposure causes protein oxidation, lipid peroxidation, oxidative DNA damage, and mitochondrial injury, thereby amplifying inflammation and impairing immune function [20]. Conversely, insufficient ROS production can compromise antimicrobial defense, demonstrating that immune homeostasis depends on tightly regulated oxidant generation.

### 2.2. ROS in Innate and Adaptive Immunity

In innate immunity, neutrophils and macrophages generate NOX2-dependent superoxide during the oxidative burst, producing downstream oxidants required for pathogen elimination [21]. In neutrophils, ROS contribute to phagocytosis, degranulation, and neutrophil extracellular trap formation, while mtROS regulate the oxidative burst, hypoxia-inducible factor 1-alpha (HIF-1α) stabilization, and inflammatory resolution [22]. Accordingly, defective ROS generation in chronic granulomatous disease and glucose-6-phosphate dehydrogenase deficiency impairs antimicrobial defense and increases susceptibility to infection [22,23].

ROS also regulates immunometabolic adaptation. Lipopolysaccharide (LPS)-stimulated M1-like macrophages shift from oxidative phosphorylation to aerobic glycolysis through pathways including the mammalian target of rapamycin (mTOR)/HIF-1α axis [24,25]. This reprogramming supports NOX- and inducible nitric oxide synthase-dependent oxidant production, whereas Nrf2, glutathione, and glutathione peroxidase 4 help control macrophage polarization, inflammatory resolution, and oxidative injury [26,27].

In adaptive immunity, T-cell receptor stimulation induces NOX- and mitochondria-derived ROS, enhancing calcium signaling, nuclear factor of activated T cells activation, interleukin-2 production, and downstream mitogen-activated protein kinase (MAPK), nuclear factor kappa-light-chain-enhancer of activated B cells (NF-κB), and mTOR pathways [28]. Mitochondrial complex III-derived ROS are also required for antigen-specific T-cell activation and clonal expansion [29]. However, sustained ROS production can disrupt these pathways, impair mitochondrial function, and promote lymphocyte apoptosis [30]. Single-cell studies further reveal distinct redox signatures associated with CD8^+^ T-cell activation, hypoxia, exhaustion, and chimeric antigen receptor T-cell persistence, underscoring the functional heterogeneity of immune redox states [31].

### 2.3. Redox-Sensitive Inflammatory Pathways

Major redox-sensitive inflammatory pathways include NF-κB, MAPKs, Janus kinase/signal transducer and activator of transcription, phosphoinositide 3-kinase–protein kinase B–mTOR, HIF-1α, and inflammasome signaling [4,13]. Through reversible amino acid oxidation and regulation of phosphatases and kinases, ROS connect cellular metabolism with immune activation. Their effects depend on the oxidant source, intensity, duration, and cellular context, allowing them to amplify or restrain inflammation [32].

NF-κB and MAPKs are central redox-regulated hubs through which ROS promote kinase activation, cytokine production, inflammatory gene expression, and stress responses [33]. Under chronic oxidative stress, mtROS can establish feed-forward loops that sustain inflammation and cellular dysfunction [20,32]. These effects are counterbalanced by the Kelch-like ECH-associated protein 1 (KEAP1)–Nrf2 pathway, which induces antioxidant, detoxification, and cytoprotective genes while limiting pro-inflammatory cytokine production [34,35].

The nucleotide-binding oligomerization domain-, leucine-rich repeat-, and pyrin domain-containing protein 3 inflammasome is also regulated by mitochondrial dysfunction, oxidized mitochondrial DNA, and ROS-dependent signals. Its activation promotes interleukin-1β and interleukin-18 maturation and induces pyroptotic cell death through cleavage of gasdermin D (GSDMD) into its pore-forming N-terminal fragment (N-GSDMD). The interaction between Nrf2 and inflammasome signaling varies according to the stimulus and cellular context, illustrating the dual role of redox pathways in inflammation [36].

**Figure 1:**
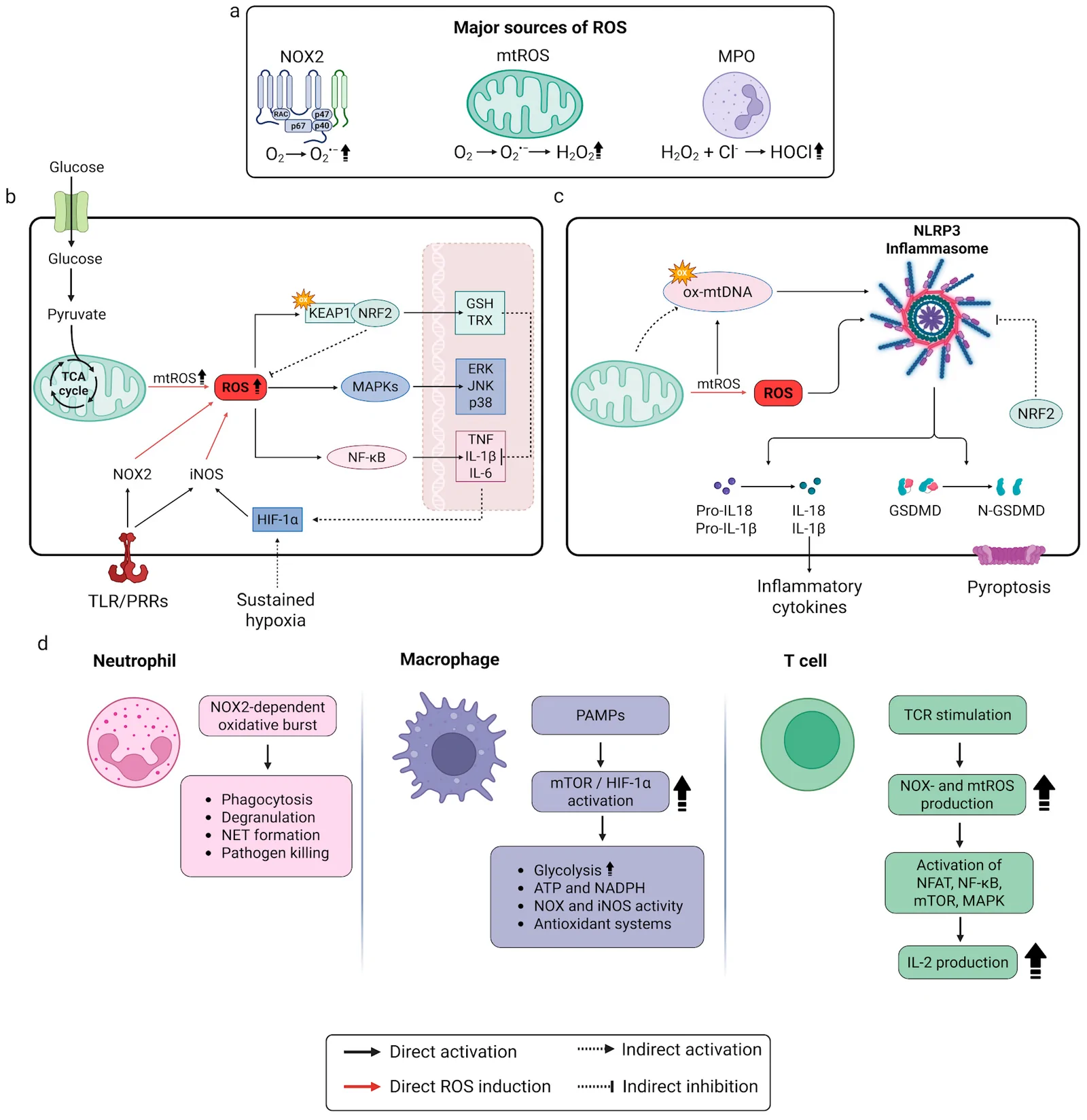
Redox-sensitive pathways linking oxidative metabolism to immune function. (**a**) Major intracellular sources of ROS, including NOX2, mtROS, and MPO. (**b**) Overview of redox-sensitive inflammatory signaling pathways. ROS generated downstream of PRRs and mitochondrial metabolism regulate NF-κB, MAPKs, HIF-1α, and the KEAP1-Nrf2 pathway, integrating inflammatory signaling with antioxidant responses. (**c**) Redox-dependent regulation of the NLRP3 inflammasome. Mitochondrial dysfunction, mtROS, and ox-mtDNA promote inflammasome activation, leading to IL-1β and IL-18 maturation, gasdermin D cleavage, and pyroptosis. (**d**) Representative redox-dependent mechanisms in innate and adaptive immunity. In neutrophils, NOX2-derived ROS mediate the oxidative burst required for phagocytosis, degranulation, NET formation, and pathogen killing. In macrophages, PAMPs induce metabolic reprogramming through the mTOR–HIF-1α axis, increasing glycolysis and NADPH availability to support NOX and iNOS activity. In T cells, TCR stimulation induces NOX- and mitochondrial-derived ROS, promoting activation of NFAT, NF-κB, MAPKs, mTOR, and IL-2 production (Created in BioRender. Silva, L. (2026) https://BioRender.com/jhsvgc8). ATP: adenosine triphosphate; ERK: extracellular signal-regulated kinase; GSDMD: gasdermin D; N-GSDMD: N-terminal fragment of GSDMD; GSH: reduced glutathione; HIF-1α: hypoxia-inducible factor 1 alpha; IL-18: interleukin-18; IL-1β: interleukin-1 beta; IL-2: interleukin-2; IL-6: interleukin-6; iNOS: inducible nitric oxide synthase; JNK: c-Jun N-terminal kinase; KEAP1: Kelch-like ECH-associated protein 1; MAPKs: mitogen-activated protein kinases; MPO: myeloperoxidase; mTOR: mechanistic target of rapamycin; mtROS: mitochondrial reactive oxygen species; mtROS: mitochondrial ROS; NADPH: nicotinamide adenine dinucleotide phosphate: NET: neutrophil extracellular trap; NFAT: nuclear factor of activated T cells; NF-κB: Nuclear factor kappa-light-chain-enhancer of activated B cells; NLRP3: Nucleotide-binding oligomerization domain-, leucine-rich repeat-, and pyrin domain-containing protein 3; NOX2: NADPH oxidase 2; Nrf2: nuclear factor erythroid 2–related factor 2; ox-mtDNA: oxidized mitochondrial DNA; PAMPs: pathogen-associated molecular patterns; PRRs: pattern recognition receptors; ROS: reactive oxygen species; TCA: tricarboxylic acid; TCR: T cell receptor; TLR: toll-like receptor; TNF-α: tumor necrosis factor-alpha; TRX: thioredoxin.

Together, these pathways integrate oxidative metabolism, mitochondrial function, and immunity to maintain homeostasis (Figure 1). Microbial metabolites and host–microbiota interactions can further modulate mitochondrial metabolism, ROS production, antioxidant defenses, and inflammatory signaling, establishing the MMA as an important interface in immunoredox regulation [37].

## 3. The Microbiota–Mitochondria Axis in Immunoredox Regulation

The evolutionary link between the microbiota and mitochondria lies in their shared ancestry. According to the endosymbiotic theory proposed by Lynn Margulis (1967), mitochondria originated from ancient α-proteobacteria engulfed by primitive eukaryotic cells. Consistent with this shared origin and their structural, genetic, and functional similarities, communication within the MMA involves mechanisms such as SCFA signaling and the regulation of oxidative stress, both of which are severely disrupted during dysbiosis [8].

SCFAs, such as acetate, propionate, and butyrate, act as key mediators of the MMA. Following cellular uptake, SCFAs can be metabolized within mitochondria, contributing to ATP production through oxidative phosphorylation. Among these, butyrate serves as the primary energy substrate for colonocytes [38]. Its oxidation drives high rates of mitochondrial oxygen consumption, reducing local epithelial oxygen levels and establishing physiological hypoxia at the mucosal surface. This anaerobic microenvironment restricts the expansion of facultative anaerobic pathogens and preserves the niche required by obligate anaerobes to maintain intestinal homeostasis [39].

Beyond their role as energy substrates, SCFAs function as signaling molecules that activate AMP-activated protein kinase (AMPK), which subsequently upregulates peroxisome proliferator-activated receptor gamma coactivator 1-alpha (PGC-1α), a key regulator of mitochondrial biogenesis. Activation of the AMPK/PGC-1α pathway promotes mitochondrial biogenesis, preserves the epithelial mitochondrial network, and supports energy production and mucosal barrier integrity [40]. This pathway also contributes to HIF-1α stabilization by enhancing mitochondrial oxygen consumption and maintaining the epithelial hypoxia required to prevent its proteasomal degradation [41]. Activated HIF-1α reinforces mucosal barrier function and helps sustain the physiological hypoxia necessary for intestinal homeostasis [42].

Failure of this homeostatic mechanism allows oxygen to enter the intestinal lumen, disrupting the niche of obligate anaerobes and promoting dysbiosis [43]. As SCFA levels decline, reduced mitochondrial respiration favors the expansion of facultative anaerobic Gram-negative bacteria and increases the luminal LPS burden. The resulting reduction in energy substrate availability for colonocytes, combined with elevated endotoxin exposure, compromises intestinal barrier function and downregulates key tight junction proteins, including claudin-1, occludin, and zonula occludens-1 [44].

As barrier integrity deteriorates, LPS translocates across the epithelium and binds to Toll-like receptor 4 (TLR4) on mucosal and immune cells. TLR4 activation induces pro-inflammatory signaling, leading to increased mtROS production and reduced mitochondrial membrane potential [45]. Consequently, colonocyte mitochondria exposed to both reduced availability of substrates for oxidative metabolism and LPS-driven inflammatory signals progressively lose bioenergetic efficiency and become important sources of cellular oxidative stress [46].

When innate mechanisms fail to restore intestinal homeostasis, mitochondrial redox signaling contributes to the transition from localized epithelial dysfunction to systemic immune responses through the gut-associated lymphoid tissue (GALT). Under homeostatic conditions, an intact intestinal barrier limits GALT exposure to luminal antigens [47]. Dysbiosis, however, compromises barrier integrity and facilitates the translocation of microbial products across the epithelium [48]. Upon reaching the lamina propria, these molecules alter the tolerogenic immune environment of the GALT and influence T-cell receptor activation in resident naïve T lymphocytes [49].

Following antigen recognition, T cells undergo metabolic reprogramming characterized by increased mitochondrial activity and a regulated increase in mtROS production, particularly from respiratory complexes I and III [50]. Rather than primarily causing oxidative damage, mtROS-derived H_2_O_2_ functions as a signaling intermediate that supports T-cell survival and clonal expansion. By modulating intracellular redox thresholds, mtROS promotes signaling pathways such as MAPK and NF-κB, which support IL-2 transcription and T-cell differentiation [51].

Persistent gut dysbiosis promotes a mitochondria-dependent immunometabolic shift within the GALT, favoring the differentiation of naïve T cells into pro-inflammatory helper subsets, particularly Th1 and Th17 cells. Sustained mitochondrial redox signaling and metabolic reprogramming support the persistence of these effector populations and reinforce local inflammation. Their migration from the intestinal mucosa into the systemic circulation may contribute to a chronic low-grade inflammatory state beyond the gut [52].

**Figure 2:**
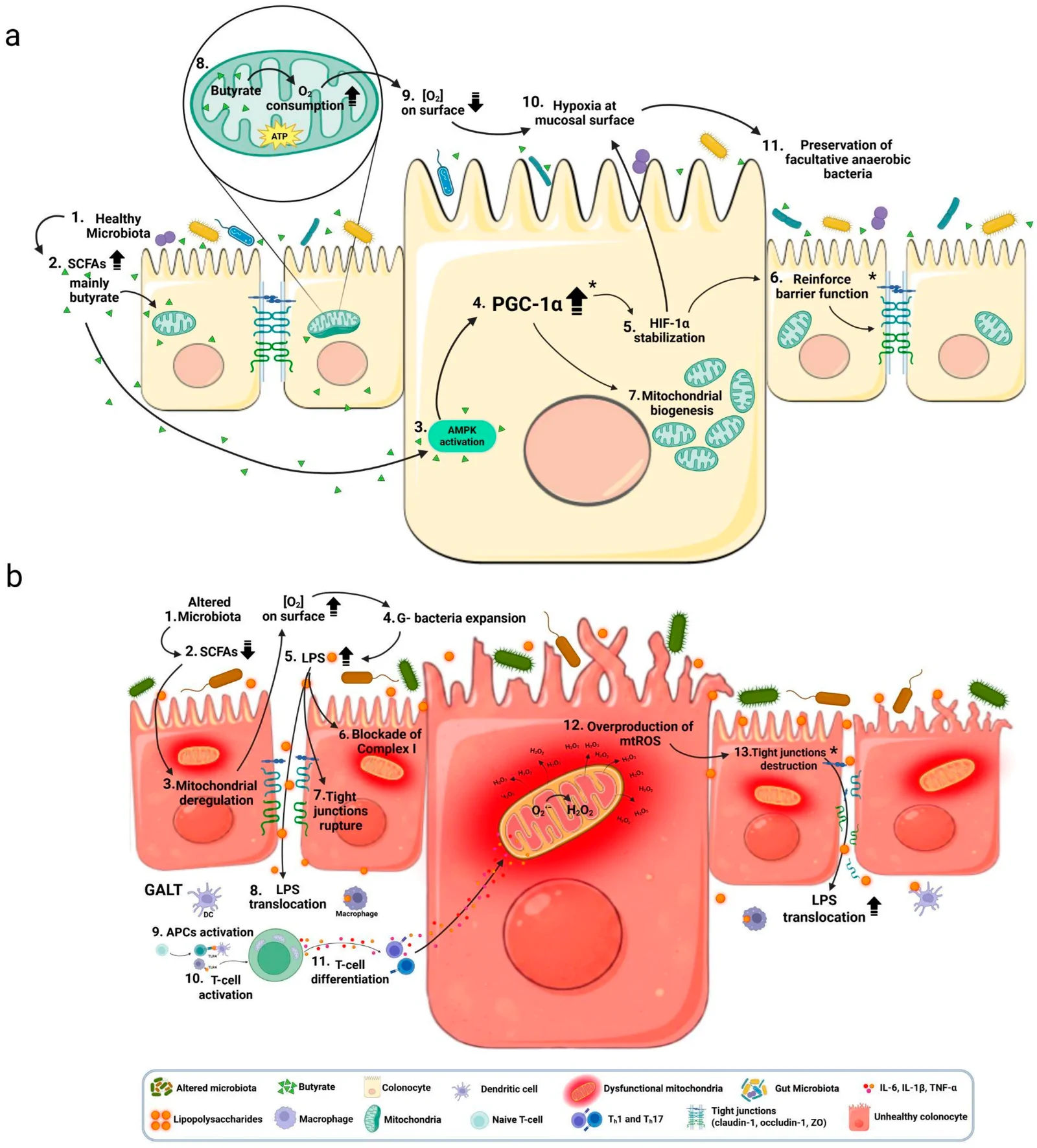
Metabolic interactions at the intestinal epithelium: from physiological hypoxia to dysbiosis-induced inflammation. (**a**) Under physiological conditions, a healthy gut microbiota (1) ferments dietary fibers to produce SCFAs, mainly butyrate (2). Butyrate is absorbed by colonocytes, leading to AMPK activation (3), which subsequently upregulates PGC-1α (4). The increase in PGC-1α activates two major pathways: it promotes HIF-1α stabilization (5), which reinforces the epithelial barrier function (6), and it stimulates mitochondrial biogenesis (7). Concurrently, butyrate metabolism within the mitochondria drives high ATP production and intense oxygen (O_2_) consumption (8). This high intracellular oxygen demand decreases the O_2_ concentration at the luminal surface (9), maintaining a state of hypoxia at the mucosal surface (10). This hypoxic environment is essential for the preservation of facultative anaerobic bacteria (11), thereby ensuring intestinal homeostasis. (**b**) Conversely, during gut dysbiosis, an altered microbiota (1) leads to a marked decrease in the production of SCFAs (2). This metabolic disruption causes mitochondrial deregulation (3), resulting in impaired colonocyte respiration and elevated O_2_ levels on the mucosal surface. This newly oxygenated environment favors Gram-negative (G-) bacterial expansion (4), leading to an increased production of LPS (5). High levels of LPS directly cause the blockade of mitochondrial Complex I (6) and initiate tight junction rupture (7). Consequently, LPS translocation (8) occurs through the compromised epithelial barrier into the GALT. Within the GALT, LPS is recognized by APCs (9), such as macrophages and dendritic cells (DCs), via Toll-like receptor 4 (TLR4). This interaction triggers naïve T-cell activation (10) and subsequent T-cell differentiation (11) into Th1 and Th17 cells. These differentiated T-cells orchestrate a massive release of pro-inflammatory cytokines (IL-6, IL-1β, and TNF-α). The influx of these cytokines, combined with the blocked mitochondrial respiratory chain, drives the overproduction of mitochondrial reactive oxygen species (mtROS), primarily hydrogen peroxide (H_2_O_2_) and superoxide (12). This severe intracellular oxidative stress leads to extensive tight junction destruction (13), severely compromising the intestinal barrier and perpetuating a vicious cycle of leaky gut and chronic inflammation (Created in BioRender. Silva, L. (2026) https://BioRender.com/t60fkot and https://smart.servier.com). * Symbolizes hypothesized pathways. AMPK: adenosine monophosphate-activated protein kinase; APC: antigen-presenting cell; ATP: adenosine triphosphate; DC: dendritic cell; GALT: gut-associated lymphoid tissue; GM: gut microbiota; HIF-1α: Hypoxia-Inducible Factor 1-alpha; IL: interleukin; LPS: lipopolysaccharides; mtROS: mitochondrial reactive oxygen species; PGC-1α: peroxisome proliferator-activated receptor-gamma coactivator 1-alpha; SCFAs: short-chain fatty acids; Th: T helper cell; TLR4: toll-like receptor 4; TNF-α: tumor necrosis factor-alpha.

Collectively, these mechanisms involving mitochondrial redox signaling demonstrate how disturbances in microbiota–mitochondria homeostasis can propagate beyond the intestinal compartment through interconnected immune, metabolic, and redox networks. Persistent inflammatory signaling and the dissemination of metabolically reprogrammed immune cells position mitochondrial dysfunction as a mechanistic link between intestinal dysbiosis and systemic immune-mediated diseases (Figure 2). Thus, the MMA represents not only a regulator of intestinal homeostasis but also an important determinant of systemic inflammatory status, providing a rationale for microbiota-targeted strategies, including probiotics, aimed at restoring mitochondrial redox balance and immune homeostasis.

## 4. Probiotic Effects on Immune Cell Redox Metabolism and Oxidative Stress Regulation

The immunomodulatory effects of probiotics are largely mediated through the MMA and depend on the immune cell population involved, probiotic strain, dose, and local microenvironment. Rather than eliciting a uniform antioxidant response, probiotics fine-tune immune cell metabolism and redox homeostasis, influencing inflammatory signaling and effector functions in a context-dependent manner. Accordingly, the following sections discuss how these mechanisms operate across the major innate and adaptive immune cell populations.

### 4.1. Macrophage and Dendritic Cell Redox Reprogramming

Macrophages constitute one of the earliest immune targets through which probiotics reshape redox homeostasis. Rather than uniformly suppressing oxidative stress, probiotic strains modulate macrophage immunometabolism according to the inflammatory context, promoting either antimicrobial ROS generation or inflammation-resolving antioxidant responses. This functional plasticity depends on strain-specific microbial signals, secreted metabolites, and receptor-mediated pathways that ultimately determine macrophage polarization [53].

Pro-inflammatory redox activation has been demonstrated with two distinct probiotic-derived stimuli. Outer membrane vesicles derived from *Escherichia coli* Nissle 1917 were phagocytosed by murine RAW264.7 macrophages during a 16 h co-incubation, triggering HIF-1, mTORC1, and NF-κB signaling, increasing intracellular ROS and the levels of TNF-α, IL-6, IL-1β, and NO, and driving M1-type polarization [54]. In a complementary study combining in vivo and in vitro experiments, BALB/c mice received *Lactobacillus plantarum* RS-09 orally (5 × 10^9^ CFU/day for 7 consecutive days) before a lethal-dose challenge with *Salmonella typhimurium* ATCC14028 (1 × 10^5^ CFU). In parallel, RAW264.7 cells were co-treated with RS-09 and the same pathogen in vitro. RS-09 enhanced ROS and NO production through TLR2/NF-κB activation in macrophages and reduced splenomegaly and mortality in the animal model [55].

In contrast, β-hydroxybutyrate (BHB), the key active metabolite of *Lacticaseibacillus rhamnosus* M2b, suppressed iNOS, CD86, IL-1β, IL-6, and TNF-α while upregulating CD163 and IL-10 in RAW264.7 macrophages stimulated with 1 mM uric acid and co-treated with 0.5 mM BHB or 10% M2b-conditioned medium for 24 h. This effect depended on GPR109a and downstream AMPK activation [56]. In another study, a probiotic mixture containing *Ligilactobacillus salivarius* H3, *Bacillus stratosphericus* J1366, and *Priestia megaterium* J1037 was administered orally to mice (3 × 10^8^ CFU every 2 days for 7 doses, starting 14 days before infection) before the induction of acute lung injury by intranasal MRSA administration (1 × 10^9^ CFU/mL). Pretreatment increased fecal and serum butyrate levels, shifted alveolar macrophages toward the M2 phenotype, and restored pulmonary GSH-Px, T-SOD, and CAT activities while lowering malondialdehyde (MDA), a marker of lipid peroxidation. It also reduced lung bacterial load, inflammatory cytokine levels, and oxidative stress markers, with efficacy comparable to that of vancomycin [57]. Together, these findings indicate that probiotic-mediated macrophage modulation involves selective regulation of redox metabolism rather than generalized suppression of oxidative pathways.

A similar pattern of regulation is observed in dendritic cells, in which probiotic-induced redox responses influence downstream adaptive immune activation. Controlled ROS production, together with changes in intracellular antioxidant capacity, appears to shape the balance between tolerogenic and inflammatory dendritic cell phenotypes. This balance depends on the exposure conditions, such that the same class of probiotic stimulus can drive dendritic cells toward either phenotype [58,59]. The following studies illustrate how these conditions influence dendritic cell redox responses and maturation.

Oral administration of *Lactobacillus rhamnosus* OLL2838 (1 × 10^10^ CFU/dose, four-day pretreatment) to mice with gliadin/indomethacin-induced gluten enteropathy restored intestinal GSH levels and glutathione S-transferase activity. These findings were consistent with the induction of low-level ROS production, IL-10, and NO in bone marrow-derived immature dendritic cells (iDCs) from the same mouse model following in vitro exposure to irradiated bacteria (1 × 10^7^ CFU/mL, 48 h co-culture) [60]. Similarly, the eight-strain formulation VSL#3 comprises four lactobacilli (*L. acidophilus*, *L. delbrueckii* subsp. *bulgaricus*, *L. casei*, and *L. plantarum*), three bifidobacteria (*B. longum*, *B. infantis*, and *B. breve*), and *Streptococcus salivarius* subsp. *thermophilus*. Exposure to this formulation at a dose equivalent to 10^8^ CFU/mL for 4 h promoted IL-10 production and suppressed Th1 responses in human blood and colonic lamina propria dendritic cells, although this activity was largely attributable to the bifidobacterial strains [61]. Probiotic effects were also dose dependent. In human monocyte-derived DCs from 14 healthy donors, 48 h exposure to UV-inactivated *Lactobacillus rhamnosus* Lcr35 maintained an immature/tolerogenic phenotype at a low bacterial concentration (10^3^ CFU/mL) but induced a semi-mature inflammatory phenotype favoring Th1/Th17 responses at a higher concentration (10^7^ CFU/mL) [58]. These observations further support the concept that microbiota-derived signals shape DC function through integrated immunometabolic and redox mechanisms.

### 4.2. Innate Mucosal and Circulating Immune Cells: Neutrophils, NK Cells, and Mast Cells

Beyond macrophages, dendritic cells, and the T-cell compartment, probiotics also influence a broader network of innate mucosal and circulating immune cells, including neutrophils, natural killer (NK) cells, and mast cells. These cells contribute to antimicrobial and antiviral defense at the intestinal barrier. The biological activities of p-hydroxycinnamic acids may also help preserve the integrity of this barrier [62]. These cell types respond to the barrier dysfunction described in Section 3: as epithelial integrity deteriorates and microbial products translocate across the mucosa, neutrophils, NK cells, and mast cells are recruited and activated, often through redox-sensitive signaling. Compared with macrophages and dendritic cells, however, the mechanisms of probiotic action in these cell types remain less well characterized, particularly with respect to the antioxidant enzymes SOD, CAT, and GPx and the transcription factor Nrf2, which are central to the immunoredox framework discussed elsewhere in this review.

Probiotic strains modulate the neutrophil oxidative burst and neutrophil extracellular trap (NET) formation in a strain- and dose-dependent manner. *Lactobacillus rhamnosus* GG (10^8^ CFU/mL) inhibited PMA- and *Staphylococcus aureus*-induced NET formation in neutrophil models, reducing ROS production and phagocytic activity while protecting against cytotoxicity. Although the authors described these effects as “antioxidative,” classical antioxidant mediators such as SOD, CAT, GPx, and Nrf2 were not directly assessed [63]. In contrast, *Bacillus subtilis* TO-A (bacteria-to-cell ratios ranging from 0.1:1 to 10:1, 1 h co-culture with nHL-60 neutrophil-like cells) regulated NET formation in a bidirectional, dose-dependent manner. Low bacteria-to-cell ratios promoted NET release, whereas higher ratios suppressed it through bacterial acid and glucose consumption rather than classical antioxidant pathways [64]. Strain identity also determined which innate cell type was preferentially affected. Mice received fermented milk containing *Lactobacillus helveticus* NCDC292, *L. acidophilus* NCDC15, or *L. paracasei* for 60 days, with all preparations standardized to 1 × 10^9^ cells/mL. *L. helveticus* most effectively enhanced neutrophil respiratory burst-associated enzyme activities (cytochrome c reductase and myeloperoxidase), whereas *L. paracasei* more strongly enhanced macrophage function in the same animals [65].

Probiotic-mediated modulation also extends to NK cells and mast cells, although direct evidence linking these effects to classical antioxidant enzymes remains lacking. *Lacticaseibacillus casei* K11 (5 × 10^6^ CFU/mL in vitro; 5 × 10^8^ CFU/mL in vivo) enhanced NK cell cytotoxicity by increasing perforin and granzyme B secretion through NKp30/NKp46-mediated ERK activation, with lipoteichoic acid identified as the active bacterial component [66]. This finding identifies a bacterial cell wall component, rather than a secreted metabolite or redox mediator, as the active signal, suggesting a mechanism more closely aligned with classical pathogen-associated molecular patterns (PAMP) recognition than with the immunoredox processes emphasized elsewhere in this review. Consistent with these findings, oral administration of *Bacillus coagulans* SNZ 1969 (5 × 10^9^ CFU/day for 12 weeks) increased NK cell activity and serum and salivary IgA levels in a randomized, placebo-controlled clinical trial [67]. In human blood-derived mast cells, co-culture with *Lactobacillus rhamnosus* GG and Lc705 (bacteria-to-cell ratio of 5:1, 3 or 24 h) downregulated the high-affinity IgE receptor (FCεR1) and histamine H4 receptor (HRH4), suggesting that specific probiotic strains may also attenuate allergic activation [68].

None of the studies reviewed in this subtopic directly evaluated immunoredox mechanisms, including antioxidant enzyme activity (SOD, CAT, GPx), Nrf2 signaling, mtROS generation, mitochondrial function, or broader immunometabolic pathways in neutrophils, NK cells, or mast cells. This represents a major knowledge gap, given the central role of these mechanisms in probiotic-mediated immunoredox regulation of macrophages and dendritic cells. Whether microbiota-dependent mitochondrial pathways and redox mechanisms described in macrophages and dendritic cells, including SOD/CAT/GPx induction, regulation of the Nrf2–KEAP1 pathway, and metabolic reprogramming by probiotic-derived metabolites, also govern neutrophil, NK cell, and mast cell responses remains largely unexplored.

### 4.3. T-Cell Compartment: Treg/Th17 Balance and Redox-Sensitive Differentiation

The CD4^+^ T-cell compartment represents another major target through which probiotics exert immunoredox control, distinct from the mechanisms described above in myeloid cells. Here, probiotics act at two convergent levels: direct engagement of pattern-recognition receptors that shifts the Treg/Th17 balance, and metabolic reprogramming of T-cell mitochondrial respiration during activation and clonal expansion. As with the innate cells discussed in Section 4.2, the effects on antioxidant enzymes remain poorly investigated in this compartment. This gap is particularly notable because GPx4-dependent suppression of ferroptosis is a well-established protective mechanism in Treg cells [69], yet its modulation by defined probiotic strains remains to be established.

Specific probiotic strains shape the Treg/Th17 balance through pattern-recognition receptor signaling. Extracts prepared by ultrasonication of *Lactobacillus rhamnosus* GG (LGG, strain CICC 6141) and the periodontal pathogen *Porphyromonas gingivalis* (ATCC 33277) were used to stimulate murine CD4^+^ T cells in vitro. At 50 µg/mL, identified as the lowest effective concentration for both extracts in preliminary dose–response experiments, LGG extract decreased the proportion of IL-17^+^ Th17 cells and increased that of CD25^+^Foxp3^+^ Treg cells through TLR2 signaling. In contrast, *P. gingivalis* extract at the same concentration induced the opposite shift through TLR4. In vivo, stimulated CD4^+^ T cells were adoptively transferred intravenously (1 × 10^6^ cells/mouse) two days before the induction of DSS-induced colitis (3% DSS; mice were sacrificed on day 8). LGG-costimulated cells reduced the Th17/Treg ratio and alleviated colitis severity through the JAK–STAT pathway, whereas *P. gingivalis*-stimulated cells exacerbated colitis [70]. SOD, CAT, GPx, and Nrf2 were not assessed in this study.

Strain specificity extends even within a single species. Two *Bifidobacterium adolescentis* strains with similar adhesion capacities but different aggregation phenotypes, the non-aggregating IF1-03 and the auto-aggregating IF1-11, were co-cultured in vitro with RAW264.7 macrophages (5 × 10^5^–10^7^ CFU/mL, 24 h) and murine splenocytes (1 × 10^7^ CFU, splenocyte-to-bacteria ratio of 5:1, 72 h). The strains were also administered intragastrically to mice with DSS-induced colitis (5 × 10^8^ CFU/day for 15 days). IF1-03 and its purified exopolysaccharide promoted IL-10 production and Treg differentiation through TLR2–ERK/p38 MAPK signaling in macrophages and protected against colitis in vivo, whereas IF1-11 favored IL-6 secretion and Th17 induction under the same conditions [71]. These findings indicate that Treg/Th17 outcomes are strain-specific and depend on the microbial signals and receptor-mediated pathways involved.

Beyond receptor engagement, gut redox mediators help translate microbial signals into T-cell fate decisions. A narrative review of the literature on gut redox signaling and T cells identified NO as a central regulator with dual effects on this balance: NO can promote pro-inflammatory Th17 differentiation and exacerbate intestinal inflammation, but it can also support Treg expansion, depending on its local concentration and the redox context [72]. The same review reports that probiotic and commensal microorganisms produce antioxidant metabolites, such as folate and glutathione, and antioxidant enzymes, including SOD and CAT, and modulate T-cell redox status through MAPK, protein kinase C (PKC), and Nrf2 signaling. Whether specific probiotic strains use this NO-dependent regulatory mechanism to preferentially expand Treg populations remains unresolved. It also remains unclear whether defined probiotic strains directly modulate GPx4-dependent protection of Treg cells against lipid peroxidation and ferroptosis.

Microbiota-derived metabolites also directly reprogram T-cell mitochondrial metabolism during activation and clonal expansion. The tryptophan-derived microbial metabolite indole-3-propionic acid has emerged as an important mediator linking microbial metabolism to adaptive immune function because of its ability to regulate cellular bioenergetics and inflammatory responses. In experimental models, indole-3-propionic acid enhanced mitochondrial respiration in murine and human CD4^+^ T cells by increasing fatty acid and amino acid oxidation while suppressing glycolytic capacity through peroxisome proliferator-activated receptor beta/delta (PPAR-β/δ) activation. This metabolic shift selectively inhibited Th1/Th17 differentiation while preserving Treg cells. Adoptive transfer experiments confirmed that these effects were T-cell-intrinsic and resulted in reduced colitis severity [73]. These findings are consistent with the broader principle that mitochondrial complex III-derived ROS are required for antigen-specific T-cell activation and IL-2 induction in CD8^+^ T cells stimulated in vitro [74]. Together, they suggest that probiotic-derived metabolites capable of modulating mitochondrial respiratory capacity may provide a means of regulating T-cell clonal expansion.

### 4.4. Central Nervous System-Immune Effectors: Microglia and Astrocytes

In addition to the peripheral and mucosal immune compartments discussed above, probiotics influence resident cells with immune functions in the central nervous system (CNS), including microglia and astrocytes, through the microbiota–gut–brain axis, where polyphenols can alter the abundance of microbiota associated with neuroinflammation [75]. Microglia serve as resident macrophages in the CNS and share much of the redox machinery described in Section 4.1, as gut microbe-mitochondria crosstalk may regulate the function and development of microglia [38], whereas astrocytes provide complementary neuroinflammatory and antioxidant regulation. Because neuroinflammation and oxidative stress in glial cells are central to the pathophysiology of neuropathic pain (NP), the focus of Section 5.1, this subsection provides the mechanistic foundation for that discussion. Compared with the limited evidence on SOD, CAT, GPx, and Nrf2 in neutrophils, NK cells, mast cells, and T cells, the literature on CNS glia is more developed. However, the reported effects vary in magnitude and direction according to the probiotic strain and experimental model, and at least one study detected no antioxidant effect.

**Microglia.** Among CNS-resident immune cells, the strongest evidence for probiotic-mediated immunoredox regulation comes from microglia, with the clearest mechanistic insights emerging from in vitro studies. Cell-free supernatants from three gut-derived lactobacilli (*Lactiplantibacillus plantarum* Lp10, *Limosilactobacillus reuteri* Lr13, and *Lacticaseibacillus rhamnosus* Lrh19) consistently enhanced Nrf2 nuclear activity and upregulated downstream antioxidant targets (heme oxygenase-1, glutathione S-transferase, SOD, GPx, and CAT) in human HMC3 microglia, although the magnitude of SOD1 induction was strain-dependent [76]. This strain-specific pattern was reinforced by a second study in which two *L. plantarum* strains, LpJ5 and LpJ8, produced comparable reductions in NO, yet only LpJ5 increased glutathione and glutathione peroxidase. These findings indicate that anti-inflammatory activity and antioxidant enzyme induction are not necessarily coupled, even within the same species [77]. Supporting these in vitro findings, cell-free supernatants from *L. plantarum* ST-III restored hippocampal Nrf2 and SOD2 expression in vivo, accompanied by reduced endoplasmic reticulum stress and improved cognitive performance following alcohol-induced injury. These observations are consistent with a broader role for AMPK and sirtuins in mitochondrial biogenesis and adaptive responses to microbial signals [9,78].

Despite these largely consistent findings, at least one study reported a contrasting result. In 3xTg-AD mice, the multi-strain probiotic Lab4b improved cognitive performance and reduced systemic and brain inflammatory cytokine levels without altering microglial cell numbers or hippocampal oxidative stress markers [79]. These findings indicate that behavioral and anti-inflammatory benefits do not necessarily coincide with detectable activation of microglial antioxidant defenses. Probiotic-induced Nrf2/SOD signaling in vivo therefore appears to depend on factors such as strain identity, disease context, and possibly treatment duration, rather than representing a universal mechanism of action.

**Astrocytes.** Compared with microglia, evidence for probiotic-mediated immunoredox regulation in astrocytes remains more limited and less consistent. In a 6-OHDA mouse model, oral administration of the probiotic mixture LLH135 (*Limosilactobacillus fermentum* LH01, *L. reuteri* LH03, and *Lactiplantibacillus plantarum* LH05) for 4 weeks restored total SOD and mitochondrial SOD (SOD2) activities, reduced levels of the DNA oxidation marker 8-OHdG, and attenuated glial fibrillary acidic protein immunoreactivity. These findings suggest that improved antioxidant defenses were accompanied by reduced astrocyte reactivity [80]. Similarly, screening of gut-derived bacterial strains identified *Parabacteroides distasonis* MRx0005 and *Megasphaera massiliensis* MRx0029 as potent suppressors of astrocytic IL-6 production, an effect associated with their intrinsic antioxidant properties and distinct SCFA profiles. These strains have been investigated as candidates for live biotherapeutic development rather than as conventional probiotic formulations [81].

Taken together, these studies indicate that CNS glia constitute additional cellular targets through which the MMA regulates oxidative stress and neuroinflammation. However, although activation of antioxidant defenses involving SOD, CAT, GPx, and Nrf2 is frequently, but not consistently, observed in microglia, comparable effects in astrocytes are more heterogeneous and appear to depend on the probiotic strain, disease model, and specific glial endpoint examined. These findings provide a mechanistic framework linking microbiota-derived immunoredox modulation to CNS homeostasis and inform the discussion of how probiotic-mediated regulation of glial redox signaling may influence NP and other disorders.

## 5. Therapeutic Implications in Oxidative and Immune-Mediated Disorders

Neuropathic pain and autoimmune diseases, including multiple sclerosis, systemic lupus erythematosus, type 1 diabetes and rheumatoid arthritis were selected based on national study conducted using DATASUS data. Within the Brazilian Unified Health System (SUS), hospitalizations for disorders of nerves, nerve roots and plexuses (ICD-10 codes G50–G59) increased by 195.16% between 2010 and 2025. In addition, deaths attributed to systemic lupus erythematosus (ICD-10 code M32) increased by 29.03%, while hospitalizations for multiple sclerosis (ICD-10 code G35) rose by 458.28%. Hospital morbidity due to type 1 diabetes mellitus (ICD-10 code E10) increased by 3.14% between 2015 and 2025. Over the same period, hospitalizations for rheumatoid arthritis and other inflammatory polyarthropathies (ICD-10 codes M05–M14) increased by 8.09% [82].

### 5.1. Neuropathic Pain

NP is characterized by persistent pain arising from a lesion or dysfunction of the somatosensory nervous system, in which neuroinflammation, oxidative stress, and mitochondrial dysfunction interact to sustain neuronal hyperexcitability [83,84,85,86,87]. Nerve injury activates immune and glial cells, generating pro-inflammatory cytokines and an excessive ROS loop that sustains nociceptive sensitization. This cascade of events is closely linked to the MMA, wherein the disruption of microbial homeostasis and reduced SCFA production promote endotoxemia (LPS), central microglial/astrocytic activation, NOX2 upregulation, and antioxidant depletion (SOD2, CAT), as observed across both preclinical models and clinical studies of NP [88,89,90,91,92,93,94,95]. These changes may impair mitochondrial bioenergetics, marked by reduced ATP production, impaired complex III respiration, and decreased PGC-1α activation [87,88,89,93,96].

Although the MMA is increasingly implicated as a potential driver of NP pathogenesis, direct assessments of neuronal mitochondrial function following probiotic therapy remain scarce. A recent patent prospecting study highlighted the growing technological landscape of probiotic-based formulations for NP, identifying antioxidant and anti-inflammatory pathways as primary targeted mechanisms in developing pharmaceutical applications [97]. Meanwhile, a review has compiled significant evidence demonstrating that probiotic interventions can suppress systemic and central oxidative stress [12]. Together, these findings provide a rationale to investigate whether these responses extend to mitochondrial regulation. Table 1 summarizes recent studies investigating these immunoredox effects.

Oral treatment with *Lactiplantibacillus plantarum* LP28, administered pre- and post-NP model induction in rats, increased serum SCFA levels and induced macrophage recruitment and polarization toward the M2 phenotype, alongside a reduction in pro-inflammatory cytokines within the injured nerve [98]. In diabetic neuropathy models, a *Lactobacillus* pool reduced serum pro-oxidant markers (MDA) and pro-inflammatory cytokines, while restoring SOD and GPx activities in rats [99]. Similarly, equivalent doses of the *L. acidophilus* LA85 strain attenuated IL-1β levels and spinal cord oxidative stress by reducing nitrite and MDA levels, accompanied by increased expression of SOD, CAT, GPx, and Nrf2 in mice [100]. This neuroimmune and redox modulation was reflected in the attenuation of nociceptive behaviors, reducing thermal and/or mechanical hyperalgesia.

In addition to systemic redox restoration, probiotics directly target central neuroinflammation by suppressing glial reactivity. In a spinal cord injury model, *Lacticaseibacillus paracasei* Ž2 treatment suppressed microglial, astrocytic, and macrophage activation at the lesion site, as evidenced by the downregulation of CD11b, CD68, IL-6, iNOS, and C1q, alongside the inhibition of the TLR/NF-κB signaling pathway [101]. Similarly, in a painful diabetic neuropathy model, combined administration of *Lactobacillus* and *Bifidobacterium* species attenuated gut dysbiosis, preserving colonic epithelial integrity and mitigating systemic inflammation, as demonstrated by histopathological analysis and marked reductions in serum LPS and pro-inflammatory cytokines. This barrier protection likely converges with the suppression of metabolic endotoxemia, since reduced circulating LPS prevents TLR4/MyD88/NF-κB pathway activation directly within the sciatic nerve, thereby attenuating neuroinflammatory signaling and mechanical and thermal nociception [102].

Overall, preclinical evidence indicates that probiotic interventions employing predominantly *Lactobacillus* and *Bifidobacterium* species at daily doses ranging from 10^9^ to 10^10^ CFU exert potentially beneficial immunoredox, neuroprotective, and barrier-restorative effects in NP models [98,99,100,101,102]. Despite these preclinical indications of potential immunoredox and neuroprotective effects, the underlying mitochondrial mechanisms remain poorly investigated in NP. Current studies have not directly examined whether the observed antioxidant and anti-inflammatory outcomes of probiotic interventions are linked to microbiota-dependent preservation of neuronal mitochondrial bioenergetics or are secondary to reduced systemic inflammation and barrier restoration. Future studies should integrate behavioral and neuroimmune biomarkers with mechanistic analyses, such as mitochondrial respiration and PGC-1α-driven biogenesis to determine whether probiotic-induced bioenergetic recovery directly contributes to neuroprotection and antinociception in NP.

### 5.2. Autoimmune Diseases

**Multiple Sclerosis.** Redox status is increasingly recognized as an important component of different autoimmune diseases, including multiple sclerosis (MS), closely interacting with chronic inflammation and contributing to disease progression [103]. This is underscored by the use of fumarate-based disease-modifying therapies in MS, including dimethyl fumarate, monomethyl fumarate, and diroximel fumarate, which engage the Nrf2 antioxidant pathway as part of their mechanisms [104]. The interaction between oxidative stress and immune dysregulation provides a strong rationale for therapeutic strategies capable of simultaneously targeting redox and inflammatory pathways [105]. However, evidence supporting the immunoredox effects of probiotics in MS remains scarce, with only a limited number of studies directly evaluating both oxidative and immune outcomes.

Table 1 presents the current clinical evidence on the effects of probiotic supplementation on oxidative damage and systemic immune responses in MS. In a 16-week randomized, double-blind, placebo-controlled trial involving 48 participants (24 per group), Salami et al. (2019) found that probiotic supplementation reduced plasma MDA and 8-OHdG levels, decreased IL-6 and high-sensitivity C-reactive protein (hs-CRP) levels, and increased IL-10 and NO levels [106]. These findings support a potential link between probiotic-induced redox modulation and the attenuation of systemic inflammation in MS. Meta-analyses have reported improvements in selected oxidative stress and inflammatory biomarkers following probiotic supplementation in MS; however, the limited and heterogeneous evidence remains inconsistent and precludes robust conclusions [107,108].

Individual studies have likewise reported improvements in antioxidant and inflammatory markers that did not differ significantly from the placebo group [109,110]. In a 16-week randomized, double-blind, placebo-controlled trial involving 55 participants (26 in the probiotic group and 29 in the placebo group), Mohy et al. (2026) [109] found significant improvements in GPx, SOD, and GSH, together with reduced MDA levels compared to baseline, but these changes did not differ significantly from those observed in the placebo group. Total antioxidant capacity (TAC) and CAT showed no change [109]. Similarly, Asghari et al. (2023) [110] analyzed 40 participants (20 per group) in a four-month double-blind, placebo-controlled trial. Placebo capsules matched the probiotic capsules in appearance, taste, and smell. In this trial, supplementation with *Saccharomyces boulardii* increased TAC and significantly decreased hs-CRP levels [110].

Although the three trials were broadly comparable in design, their sample sizes and reporting of participant attrition varied. One study reported a dropout rate of 38.9% [109], whereas attrition was not explicitly reported in the other two studies [106,110]. These methodological differences, together with the relatively small study populations, should be considered when interpreting the consistency and strength of the clinical evidence.

Despite these clinical indications of a potential immunoredox effect, the underlying mechanisms remain poorly investigated. Clinical studies have not directly examined whether the observed antioxidant effects are linked to microbiota-dependent regulation of mitochondrial metabolism or are secondary to reduced systemic inflammation. Future studies should integrate clinical biomarkers with mechanistic analyses of mitochondrial function, immunometabolism, and redox signaling to determine whether probiotic-induced antioxidant effects directly contribute to immune regulation and neuroprotection in MS.

**Systemic Lupus Erythematosus.** Persistent oxidative stress is a hallmark of systemic lupus erythematosus (SLE) and actively contributes to immune dysregulation. T lymphocytes from patients with SLE display persistent mitochondrial hyperpolarization, cytoplasmic alkalinization, and glutathione depletion, creating a pro-oxidative intracellular environment that contributes to Th17 polarization through calcium-dependent cAMP-responsive element modulator activation [111,112]. MtROS also drives NETosis in SLE neutrophils, releasing ox-mtDNA that sustains the type I interferon signature characteristic of the disease [113].

This redox imbalance also extends to the gut, where it is closely associated with microbial dysbiosis and barrier dysfunction. In MRL/lpr lupus-prone mice, an altered Firmicutes/Bacteroidetes ratio is detectable early in disease development. By 18 weeks of age, this dysbiotic profile is accompanied by increased colonic 4-hydroxynonenal-protein adducts, loss of the tight junction protein ZO-2, and elevated fecal albumin and IgA, together with higher antinuclear and anti-smooth muscle antibody titers and more severe glomerulonephritis. Treating these mice with the antioxidant N-acetylcysteine reshapes the fecal microbiota and attenuates systemic autoimmunity, indicating that intestinal oxidative stress and dysbiosis actively sustain one another rather than simply co-occurring [114]. A comparable link is observed in patients, as serum MDA levels correlate inversely with fecal Firmicutes and Cyanobacteria abundance and positively with Actinobacteria abundance in individuals with non-active SLE [115]. Taken together, these findings support a model in which gut dysbiosis and immune-redox imbalance reinforce each other across the course of SLE, positioning both as pivotal, interacting axes of disease, and providing the rationale for microbiota-targeted strategies capable of correcting redox and immune homeostasis simultaneously [116].

Probiotic modulations have demonstrated coordinated antioxidant and immunomodulatory effects in lupus-prone murine models (Table 1), where selected *Lactobacillus* and *Bifidobacterium* strains influence antioxidant, inflammatory, and vascular pathways. In mice, oral treatment with *L. paracasei* GMNL-32, *L. reuteri* GMNL-89, or *L. reuteri* GMNL-263 significantly increased serum and hepatic GSH and 2,2-diphenyl-1-picrylhydrazyl radical-scavenging activity while reducing MDA, alongside reductions in IL-6 and TNF-α and suppression of TLR/MyD88 signaling [117]. These same strains attenuated hepatic apoptosis and inflammatory markers, including iNOS expression, matrix metalloproteinase-9 (MMP-9) activity, and CRP, via inhibition of MAPK and NF-κB signaling pathways, indicating that probiotic-mediated suppression of pro-inflammatory signaling is accompanied by reduced nitrosative stress in lupus [118].

Beyond these systemic antioxidant effects, probiotic administration has been shown to influence redox homeostasis at the vascular endothelium in lupus. In mice, *Lactobacillus fermentum* CECT5716 (LC40) reversed endothelial dysfunction associated with increased NADPH oxidase-driven superoxide production and inhibitory eNOS phosphorylation at Thr495, restoring endothelium-dependent vasodilation and lowering plasma LPS alongside proinflammatory cytokines [119]. This vasoprotective effect was reproduced in a TLR7-activated (imiquimod-induced) lupus model, where LC40 and *Bifidobacterium breve* CECT7263 normalized the pro-oxidative, pro-inflammatory vascular phenotype and restored acetylcholine-induced, NO-dependent vasorelaxation, an effect confirmed to be endothelium- and eNOS-dependent [120]. Mechanistically, this vascular redox rescue may also converge with the SCFA–Nrf2 axis, since SCFAs deficiency, notably of butyrate and acetate, has been shown to impair G protein-coupled receptor 41 and 43 signaling and suppress Nrf2-driven antioxidant enzymes such as heme oxygenase-1, thereby heightening susceptibility to oxidative damage and aggravating hypertension in the same TLR7-driven lupus model [121,122].

At the mucosal interface, direct evidence for probiotic-driven ROS scavenging has emerged from a bioengineered *L. rhamnosus* GG formulation (LGG@PDA), which markedly reduced intestinal ROS levels in lupus-prone mice, reduced the proportion of mucosal Th17 cells, and restored epithelial barrier integrity, alongside enrichment of the metabolite L-methionine, which enhanced macrophage efferocytosis in a CX3CR1-dependent manner and reduced autoantibody titers and renal pathology [123]. Also, *Bifidobacterium* and its SCFAs metabolites were shown to correct Treg, Th1, and Th17 imbalances and reduce autoantibody levels while alleviating nephritis in mice, in parallel with reduced *Bifidobacterium* abundance in patients with SLE [124]. Although this study centers on metabolite-driven immunomodulation rather than redox endpoints, the mechanistic link between SCFAs signaling and Nrf2-dependent antioxidant enzyme induction described above provides a plausible pathway through which *Bifidobacterium*-derived metabolites may act. Collectively, these studies support a model in which probiotic-mediated antioxidant responses, SCFAs signaling, and ROS/NO-dependent vascular and mucosal effects may represent complementary mechanisms linking GM modulation to immunoredox regulation in SLE [125].

**Type 1 Diabetes (T1D).** T1D is a chronic autoimmune disease driven by T-cell-mediated destruction of pancreatic β-cells, in which oxidative stress acts both as a consequence and a driver of disease progression. β-cells are intrinsically vulnerable to redox imbalance because they constitutively express lower levels of antioxidant enzymes, including SOD, CAT, and GPx, than cells in most other tissues. During insulitis, infiltrating macrophages and activated T cells release IL-1β, IFN-γ, and TNF-α, which converge on β-cell NADPH oxidase and iNOS to generate ROS/RNS that damage islet DNA, lipids, and proteins, further compromising endogenous antioxidant defenses [126]. Consistent with this vulnerability, genetic ablation of Nrf2 accelerates diabetes onset and worsens β-cell damage in chemically induced models, whereas pharmacological Nrf2 activators, such as dimethyl fumarate, delay disease onset in non-obese diabetic mice, directly paralleling the fumarate-based mechanism already discussed above for MS above [127].

This redox vulnerability is accompanied by early and disease-relevant gut dysbiosis. In a longitudinal cohort of Finnish children at high genetic risk for T1D, expansion of *Bacteroides dorei* and *Bacteroides vulgatus* was detected as early as eight months before islet autoantibody seroconversion [128]. Similarly, a case–control study found that children with two or more diabetes-associated autoantibodies exhibited reduced abundance of lactate-producing species, lower levels of *Bifidobacterium adolescentis* and *B. pseudocatenulatum*, and increased *Bacteroides* abundance compared with matched autoantibody-negative controls [129].

Direct evidence that probiotic supplementation can improve redox imbalance comes from a streptozotocin (STZ)-induced diabetic model, with a combination of *Lacticaseibacillus casei* RVSRI-4 and *Lactobacillus acidophilus* RVSRI-6 was administered to diabetic *Wistar* rats. Compared with untreated diabetic controls, probiotic administration significantly reduced serum MDA levels and increased CAT, SOD, GPx, and TAC. However, these markers were only partially restored toward healthy-control levels, and the underlying molecular mechanisms, including potential Nrf2 pathway involvement, were not directly investigated [130].

Also, in a T1D model STZ-induced but in mice, *Lacticaseibacillus paracasei* SD1 and *Lacticaseibacillus rhamnosus* SD11 preserved islet integrity and β-cell mass, while reducing macrophage and CD4^+^/CD8^+^ T-cell infiltration and suppressing iNOS and NF-κB p65, caspase-3, caspase-8, and caspase-9 activation, and upregulating anti-apoptotic Bcl2. Among the interventions tested, SD11 alone produced the most pronounced effects across the evaluated outcomes [131]. Canonical SOD/CAT/GPx activity was not directly reported in this study, though the iNOS/caspase signaling findings suggest attenuation of nitrosative and redox-linked β-cell injury.

At the clinical level, three randomized trials have investigated probiotic supplementation in relation to T1D, including studies in patients with established or new-onset disease and in individuals without T1D. In the first, 56 patients aged 6–18 years with T1D received a mixture of *Lactobacillus salivarius* subsp. *salicinius* AP-32, *L. johnsonii* MH-68, and *Bifidobacterium animalis* subsp. *lactis* CP-9, alongside standard insulin therapy [132]. In the second, 42 healthy individuals with no known T1D risk factors received *Lactobacillus johnsonii* N6.2 in a double-blind design [133]. In the third, children with new-onset T1D received a high-strength eight-strain formulation (*Streptococcus thermophilus*, four *Bifidobacterium* species, and three *Lactobacillus* species) in a randomized, double-blind, placebo-controlled design in North India [134]. All three report improvements in glycemic control (HbA1c, C-peptide), regulatory T-cell frequency, or inflammatory cytokine profiles (TGF-β1 ↑, IL-8/IL-17/TNF-α ↓). However, none directly assessed redox endpoints or microbiota-related mechanisms, including gut barrier integrity or GM-derived metabolic signals. Thus, whether probiotic-induced modulation of the MMA improves mitochondrial redox homeostasis and β-cell function in T1D remains unexplored.

The available evidence suggests that probiotics can attenuate redox and immune disturbances in experimental T1D, but the relationship between these effects and the MMA remains poorly defined. Preclinical studies indicate restoration of antioxidant defenses and attenuation of nitrosative and apoptotic signaling. Clinical trials have focused primarily on glycemic and immunological outcomes without integrating redox, microbiota, or mitochondrial endpoints. Thus, whether probiotic-induced modulation of the GM and its metabolites can improve mitochondrial redox homeostasis and preserve β-cell function remains an important unanswered question. Future studies should integrate microbiome composition, SCFA profiles, redox biomarkers, mitochondrial function, and β-cell outcomes to establish whether the MMA represents a mechanistic link between probiotic supplementation and immunoredox protection in T1D.

**Rheumatoid arthritis (RA).** RA is a prototypical autoimmune disease in which oxidative stress is not merely a consequence of joint inflammation but also an active contributor to synovial pathology. Neutrophils infiltrating the RA synovium express high levels of NOX2, a major source of local superoxide and ROS [135]. In parallel, fibroblast-like synoviocytes (FLS) exhibit mtDNA mutations and downregulation of CAT, GPx, and SOD1, indicating impaired antioxidant defense and mitochondrial redox homeostasis within the joint [136]. Increased NOX4 expression further promotes ROS-dependent FLS migration and invasion in response to IL-17 and TNF-α [137]. This redox imbalance directly interfaces with the immune response: the Nrf2/Keap1 pathway is a recognized therapeutic target in RA, with Nrf2 activation suppressing both ROS accumulation and downstream NF-κB-driven cytokine production in synovial tissue, mirroring the fumarate-Nrf2 mechanism discussed above for MS.

As in T1D, local redox vulnerability in RA is preceded by a well-characterized gut dysbiosis. In a study analyzing fecal samples from patients with new-onset untreated RA (NORA), chronic treated RA, psoriatic arthritis, and healthy controls, *Prevotella copri* was strongly and specifically associated with NORA and inversely related to *Bacteroides* abundance [138]. This dysbiotic signature has been linked to arthritis through a Th17-dependent mechanism. Germ-free SKG mice colonized with *P. copri*-dominated microbiota from RA patients, or with *P. copri* alone, developed severe synovitis following zymosan challenge, accompanied by increased intestinal Th17 cells and enhanced IL-17 responses to the arthritis-related autoantigen RPL23A in the colon and regional lymph nodes, but not spleen [139]. These findings support a functional link between *P. copri*-associated dysbiosis and intestinal immune activation in RA.

Evidence that probiotics can attenuate oxidative stress in experimental RA comes from two studies using the same combination of *Lacticaseibacillus casei* and *Lactobacillus acidophilus*. In a collagen-induced arthritis model, oral administration of live or heat-killed preparations reduced pro-inflammatory response while enhancing anti-inflammatory cytokine production. These immunomodulatory effects were accompanied by reduced MDA levels and restoration of CAT, GSH, GPx, and SOD activities in synovial exudate, with effects exceeding those of indomethacin [140]. In a complementary adjuvant-induced arthritis model, the same strains restored CAT, SOD, GSH, and GPx activities and reduced MDA levels in liver and kidney tissues, while preserving bone mineral content and organ integrity [141]. The data indicate that probiotic supplementation can modulate both local and systemic redox disturbances in experimental RA, accompanied by a shift toward an anti-inflammatory profile.

Similar antioxidant effects have been reported with *Bifidobacterium* strains. Oral administration of *B. breve* NCIM 5671, *B. longum* NCIM 5672, or *B. bifidum* NCIM 5697 reduced lipid and protein peroxidation, restored antioxidant enzyme activity, and attenuated paw inflammation while preserving bone mineral content in arthritic mice [142]. At the clinical level, the existing randomized controlled trials (RCTs) in RA patients directly reinforce this preclinical redox mechanism, in clear contrast to the T1D literature discussed above. In a Brazilian cohort of 47 RA patients, a five-strain probiotic formulation, administered for 60 days, reduced NO metabolites while increasing sulfhydryl groups and total radical-trapping antioxidant parameters [143]. Similarly, a synbiotic intervention in 54 patients with RA increased plasma NO and GSH levels and improved markers of insulin resistance [144].

RA currently represents the strongest preclinical-to-clinical evidence within this review for probiotic-mediated immunoredox modulation, with consistent improvements in antioxidant defenses and redox biomarkers. However, the mechanisms underlying these effects remain poorly defined in humans. Although GM composition and microbial-derived metabolites have been assessed in some studies, no study has directly integrated these changes with mitochondrial redox homeostasis or established a functional link across the MMA. Thus, whether the MMA connects probiotic-induced microbiota modulation to immunoredox regulation in RA remains also an important unresolved question.

Taken together, the studies reviewed suggest that probiotics may modulate redox and immune disturbances across autoimmune diseases, although the nature and depth of the evidence vary considerably. In MS, clinical studies have primarily assessed systemic oxidative stress and inflammatory biomarkers, with inconsistent findings and limited mechanistic insight. In SLE, experimental models provide a more detailed picture of vascular and intestinal redox modulation associated with changes in immune regulation and tissue injury. In T1D, antioxidant effects and attenuation of nitrosative and apoptotic signaling have been reported mainly in experimental models, whereas clinical studies have focused on glycemic and immunological outcomes without directly assessing redox responses. RA studies extend from experimental evidence of local and systemic antioxidant effects to clinical changes in circulating redox biomarkers, although the mechanisms underlying these responses remain uncertain. Thus, the apparent differences across conditions reflect not only their distinct pathological contexts but also variation in probiotic formulations, study designs, and measured outcomes, precluding conclusions about relative efficacy. Despite these differences, a shared limitation is that the causal links between probiotic-induced microbiota changes, mitochondrial redox regulation, and immune outcomes across the MMA remain to be fully established in these four disease settings.

**Figure 3:**
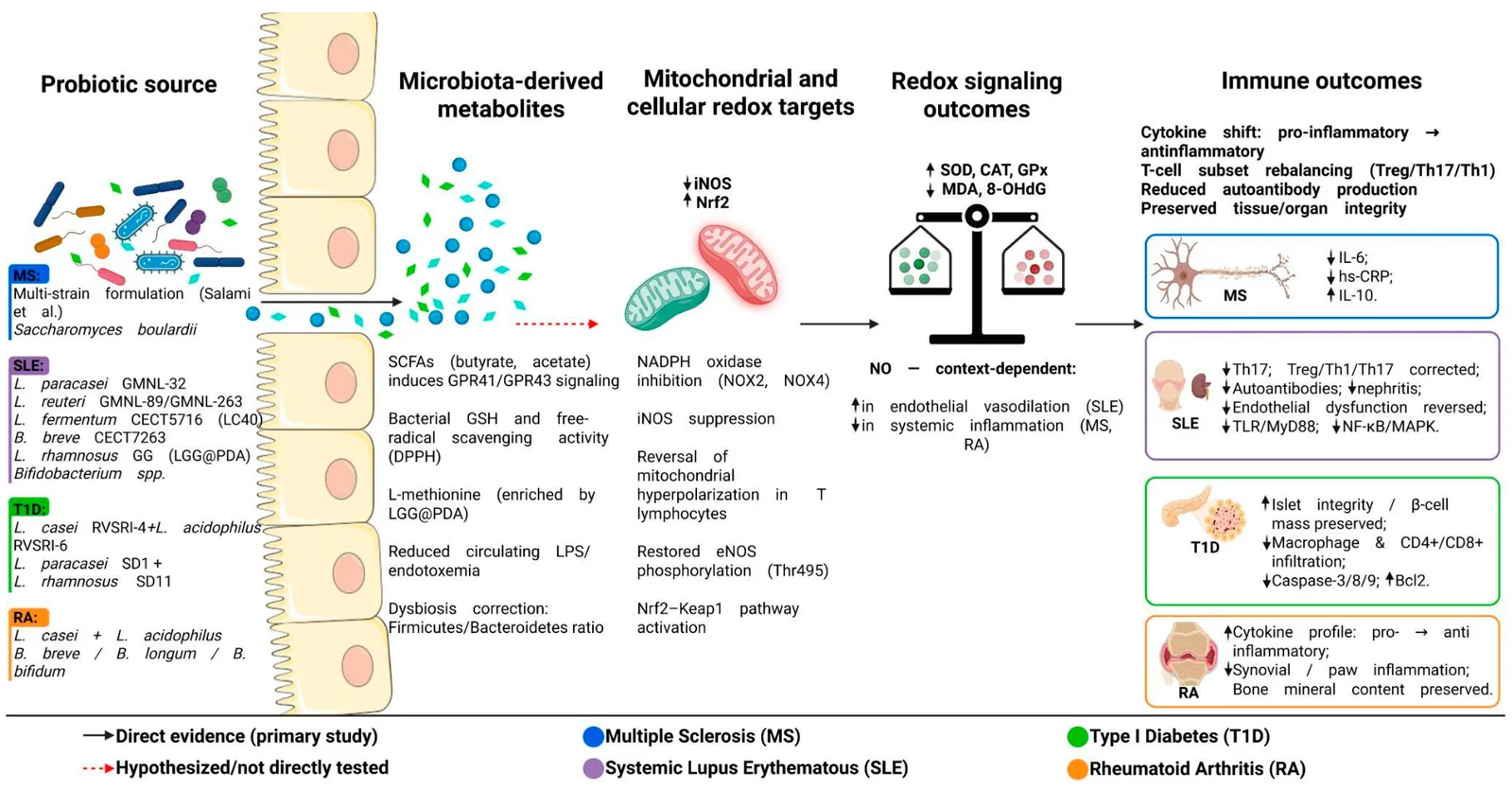
From probiotics to immune outcomes: a proposed microbiota–mitochondria–redox axis linking probiotic supplementation to immunoredox regulation across four autoimmune diseases. Schematic synthesis of the probiotic strains, metabolites, mitochondrial and cellular redox targets, and immune outcomes discussed in Section 5.2, organized as a five-step sequential model: (1) Probiotic Source—strains administered in each disease, grouped and color-coded by condition (MS [105], blue; SLE, purple; T1D, green; RA, orange); (2) Microbiota-Derived Metabolites—SCFAs, bacterial glutathione, free-radical-scavenging activity, L-methionine, and endotoxemia/dysbiosis correction reported in the reviewed studies; (3) Mitochondrial and Cellular Redox Targets—NADPH oxidase inhibition, iNOS suppression, reversal of mitochondrial hyperpolarization, eNOS restoration, and Nrf2–Keap1 pathway engagement; (4) Redox Signaling Outcomes—the direction of change in canonical antioxidant markers (SOD, CAT, GPx, GSH, TAC) and oxidative damage markers (MDA, 8-OHdG), with nitric oxide (NO) shown separately as a context-dependent marker that increases in endothelial vasodilation (SLE) but decreases in systemic inflammatory states (MS, RA); and (5) Immune Outcomes—the resulting shift in cytokine profile, T-cell subset balance, autoantibody production, and tissue integrity. The bottom strip disaggregates the immune outcomes of Step 5 into the specific findings reported for each disease. Solid arrows indicate a direct link supported by primary studies cited in the corresponding text; the dashed red arrow between Steps 2 and 3 explicitly flags that no study reviewed in this manuscript has directly tested the link between a microbiota-derived metabolite and a mitochondrial or cellular redox target within a single experimental design. This connection is inferred by juxtaposing separate lines of evidence, not demonstrated end-to-end. (Created in BioRender. Silva, L. (2026) https://BioRender.com/8sc7uof and https://smart.servier.com).

The findings on autoimmune diseases are summarized in Figure 3. This figure provides a visual summary of the key biochemical pathways, redox signaling mechanisms, and context-dependent changes in biomarkers associated with these conditions.

**Table 1:** Therapeutic immunoredox effects of probiotics across oxidative and immune-mediated disorders.

Type of Study/Species/Model	Level of Evidence	Strains	Dosage	Key Findings	Mitochondrial Endpoints	Reference
**Neuropathic Pain**						
In vivo, Sprague–Dawley rats/Chronic constriction injury of the median nerve	Preclinical	*Lactiplantibacillus plantarum* LP28	1 × 10^10^ CFU, oral, 28 days prior to 14 days post-induction	↑ Serum SCFA levels, ↑ M2 macrophage polarization and ↓ pro-inflammatory cytokines (TNF-α, IL-1β, and IL-6) within the injured nerve, and attenuated mechanical and thermal hyperalgesia	Not evaluated	[98]
In vivo, *Wistar* rats/ Diabetic neuropathy	Preclinical	*Lactobacillus rhamnosus*, *L. acidophilus*, *L. delbrueckii*, *L. plantarum*, and *Bifidobacterium bifidum*	1 × 10^9^ CFU, oral, 28 days	↓ Serum MDA levels, ↑ serum SOD and GPx activities, and attenuated mechanical and thermal hyperalgesia	Not evaluated	[99]
In vivo, C57BL/6 mice/ Diabetic neuropathy	Preclinical	*Lactobacillus acidophilus* LA85	1 × 10^9^ CFU, oral, 28 days	↓ MDA, nitrite, and IL-1β levels, ↑ SOD, CAT, GPx, and Nrf2 activities in the spinal cord, and attenuated mechanical and thermal hyperalgesia	Not evaluated	[100]
In vivo, *Wistar* rats/ Spinal cord injury	Preclinical	*Lacticaseibacillus paracasei* Ž2	5 × 10^9^ CFU/mL, oral, 7 days	↓ Markers of glial activation at the spinal cord: microglia/macrophages (CD11b), microglia (CD68, IL-6, and iNOS), and astrocytes (C1q); ↓ TLR/NF-κB signaling pathway. Pain behaviors were not evaluated	Not evaluated	[101]
In vivo, *Sprague–Dawley* rat/ Diabetic neuropathy	Preclinical	*Lactobacillus rhamnosus*, *L. plantarum*, *L. rhamnosus GG*, *L. casei*, *L. paracasei*, *L. rhamnosus*, *L. gasseri*, *L. delbrueckii* subsp. *Bulgaricus, L. reuteri 1E1*, *Bifidobacterium lactis*, *B. animalis* subsp. *lactis*, *B. breve*, *B. longum*, *B. infantis*, and *Streptococcus thermophilus*	2.4 × 10^10^ CFU/day, oral, co-administered 12 weeks during no high-fat diet/streptozotocin induction	↓ Gut dysbiosis, preserved colonic epithelial integrity, ↓ serum LPS and pro-inflammatory cytokines (TNF-α, IL-1β, and IL-6), ↓ TLR4/MyD88/NF-κB signaling in the sciatic nerve, and attenuated neuropathic pain behaviors	Not evaluated	[102]
**Multiple Sclerosis**						
RCT (double-blind); Human, MS patients	Clinical study	*L. plantarum*, *L. casei*, *L. reuteri*, *L. fermentum*, *B. lactis*, *B. infantis* (multi-strain)	2 × 10^9^ CFU/day, 16 weeks	↓ MDA; ↓ 8-OHdG; ↑ NO, SOD, and TAC. ↓ IL-6 and hs-CRP; ↑ IL-10; No change observed in GSH.	Not evaluated	[106]
Randomized, double-blind, placebo-controlled clinical trial (RCT); Human, RRMS patients	Clinical study	Lactocare^®^ (6-strain pool: *L. casei*, *L. acidophilus*, *L. rhamnosus*, *L. bulgaricus*, *B. breve*, *B. longum*, *S. thermophilus* + FOS).	2 × 10^9^ CFU, twice daily, 4 months	↑ Antioxidant enzyme activity (GPx, SOD, GSH); ↓ MDA over the treatment period; TAC and CAT remained unchanged.	Not evaluated	[109]
RCT (double-blind, placebo-controlled); Human, RRMS patients	Clinical study	*Saccharomyces* *boulardii*	1 × 10^10^ CFU, once daily, 4 months	↑ TAC with probiotic treatment; ↓ MDA. ↓ systemic inflammation, pain, and fatigue	Not evaluated	[110]
**Systemic Lupus Erythematosus**						
In vivo, NZB/W F1 mice	Preclinical (animal study)	*L. paracasei* GMNL-32, *L. reuteri* GMNL-89, *L. reuteri* GMNL-263	1 × 10^9^ CFU/day, 12 weeks	↑ GSH; ↑ radical-scavenging capacity in serum and liver; ↓ MDA; ↓ TNF-α and IL-6; ↓ TLR/MyD88 signaling; ↑ Tregs	Not evaluated	[117]
In vivo, NZB/W F1 mice	Preclinical (animal study)	*L. paracasei* GMNL-32, *L. reuteri* GMNL-89, *L. reuteri* GMNL-263	1 × 10^9^ CFU/day, 12 weeks	↓ Hepatic iNOS expression; ↓ hepatic apoptosis; ↓ inflammatory injury markers (MMP-9, CRP) through suppression of MAPK and NF-κB signaling.	Not evaluated	[118]
In vivo, NZB/W F1 vs. NZW/LacJ mice	Preclinical (animal study)	*L. fermentum* CECT5716 (LC40)	5 × 10^8^ CFU/day, 15 weeks	↓ NADPH oxidase-derived superoxide; ↓ inhibitory eNOS phosphorylation; ↑ NO-dependent vasorelaxation; ↓ disease activity, blood pressure, and cardiac/renal hypertrophy	Not evaluated	[119]
In vivo, BALB/cByJRj mice, TLR7-induced (imiquimod) model	Preclinical (animal study)	*L. fermentum* CECT5716 (LC40) and/or *Bifidobacterium breve* CECT7263 (BFM)	1 × 10^9^ CFU/day, 8 weeks	↓ Pro-oxidative vascular phenotype; ↑ NO-dependent vasorelaxation; ↓ circulating autoantibodies; ↓ splenic B-cell activation	Not evaluated	[120]
In vivo, C57BL/6J and MRL/lpr model	Preclinical (animal study)	*Polydopamine-encapsulated Lactobacillus rhamnosus GG (LGG@PDA)*	5 × 10^8^ CFU, 3 days/week	↓ Intestinal ROS; ↓ mucosal Th17 cells; ↑ macrophage efferocytosis through L-methionine; ↓ autoantibodies; ↓ renal damage	Not evaluated	[123]
**Type 1 Diabetes**						
In vivo, STZ-induced diabetic male Wistar rats, Iran	Preclinical (animal study)	*Lacticaseibacillus casei* RVSRI-4 + *Lactobacillus acidophilus* RVSRI-6	10^9^ CFU/strain/day (2 × 10^9^ CFU/rat/day total), oral, 6 weeks	Serum MDA↓ (~29%); CAT↑ (~21%), SOD↑ (~6.5%), GPx↑ (~59%), TAC↑ (~37%), only partial restoration toward healthy-control levels.	Not evaluated	[130]
In vivo, multiple low-dose STZ-induced diabetic BALB/c mice, Thailand	Preclinical (animal study)	*Lacticaseibacillus paracasei* SD1, *L. rhamnosus* SD11 (alone or combined)	10^9^ CFU/200 µL/mouse/day, oral, 8 weeks	Preserved islet integrity and β-cell mass; suppressed macrophage/CD4^+^/CD8^+^ islet infiltration; downregulated iNOS, NF-κB p65, caspase-3/8/9. SOD/CAT/GPx not directly measured.	Not evaluated	[131]
RCT (double-blind, placebo-controlled), 56 patients aged 6–18 years, Taiwan	Clinical study	*Lactobacillus salivarius* subsp. *salicinius* AP-32, *L. johnsonii* MH-68, *Bifidobacterium animalis* subsp. *lactis* CP-9	1 × 10^10^ CFU/day, 6 months	IL-8, IL-17, MIP-1β, RANTES, and TNF-α significantly reduced; TGF-β1 increased. Effects persisted 3 months post-discontinuation. No oxidative stress markers assessed.	Not evaluated	[132]
Double-blind RCT, 42 healthy individuals at no known T1D risk, USA	Clinical study	*Lactobacillus johnsonii* N6.2	1 × 10^8^ CFU/day (1 capsule), 8 weeks	Increased monocyte and NK cell numbers via IDO pathway; increased circulatory effector Th1 and cytotoxic CD8^+^ T cells; delayed memory CD8^+^ T-cell apoptosis. No oxidative stress markers assessed.	Not evaluated	[133]
Randomised, double-blind, placebo-controlled trial, children with new-onset T1D, India	Clinical study	*Streptococcus thermophilus* DSM 24731, *Bifidobacterium infantis* DSM 24737, *B. longum* DSM 24736, *B. breve* DSM 24732, *Lactobacillus acidophilus* DSM 24735, *L. plantarum* DSM 24730, *L. paracasei* DSM 24733, *L. delbrueckii* subsp. *bulgaricus* DSM 24734 (Vivomixx^®^, 8-strain formulation)	2.25 × 10^11^ CFU/day (1 sachet), oral, 6 months	Significant increase in i-Tregs (Δ+1.5 vs. −2.0 placebo) and plasma IL-10; significant fall in HbA1c, FBG, TBG, and total/bolus insulin dose; C-peptide preserved. No oxidative stress markers assessed.	Not evaluated	[134]
**Rheumatoid Arthritis**						
In vivo, collagen-induced arthritis, female Wistar rats, India	Preclinical (animal study)	*Lactobacillus casei* + *L. acidophilus* (live and heat-killed)	2 × 10^8^ CFU/mL, oral, day 1–28	Reduced IL-6, TNF-α, IL-1β, IL-17; increased IL-4, IL-10; decreased lipid peroxidation; restored CAT, glutathione, GPx, and SOD in synovial exudate, exceeding indomethacin’s effect.	Not evaluated	[140]
In vivo, adjuvant-induced arthritis, female Wistar rats, India	Preclinical (animal study)	*Lactobacillus casei* + *L. acidophilus*	2 × 10^8^ CFU/mL, oral, from day 9 post-induction	Restored CAT, SOD, reduced glutathione, and GPx in liver/kidney homogenates; lowered MDA; preserved bone mineral content (radiographically confirmed); normal organ histopathology	Not evaluated	[141]
In vivo, adjuvant-induced arthritis, male Wistar rats, India	Preclinical (animal study)	*Bifidobacterium breve* NCIM 5671, *B. longum* NCIM 5672, *B. bifidum* NCIM 5697	10^9^ CFU/mL, oral gavage, 15 days before + 15 days after induction	Reduced lipid/protein peroxidation; restored CAT, GPx, GSH reductase, and SOD; reduced paw volume; preserved bone mineral content.	Not evaluated	[142]
Randomized, double-blind, placebo-controlled trial, 47 RA patients, Brazil	Clinical study	*Lactobacillus acidophilus* La-14, *L. casei* Lc-11, *Lactococcus lactis* Ll-23, *Bifidobacterium lactis* Bl-04, *B. bifidum* Bb-06 (5-strain sachet)	10^9^ CFU/g each strain, 1 sachet/day dissolved in water before breakfast, 60 days	Reduced WBC count, TNF-α, and IL-6; reduced NO metabolites; increased sulfhydryl groups and total radical-trapping antioxidant parameters.	Not evaluated	[143]
Randomized, double-blind, placebo-controlled trial, 54 RA patients, Iran	Clinical study	*Lactobacillus acidophilus*, *L. casei*, *Bifidobacterium bifidum* (synbiotic) + inulin	2 × 10^9^ CFU/g each strain + 800 mg inulin/day, 8 weeks	Reduced hs-CRP, insulin, HOMA-IR, HOMA-B; increased plasma NO and GSH; improved DAS-28 and VAS pain	Not evaluated	[144]

Findings from preclinical models and clinical trials, highlighting key redox, immune, intestinal, and disease-related outcomes following probiotic interventions. Arrows indicate the direction of change observed after intervention: ↑ increase, ↓ decrease. 8-OHdG, 8-hydroxy-2′-deoxyguanosine; CAT, catalase; CD, cluster of differentiation; CFU, colony-forming units; CRP, C-reactive protein; DAS-28, Disease Activity Score in 28 joints; eNOS, endothelial nitric oxide synthase; FBG, fasting blood glucose; FOS, fructooligosaccharides; GSH, reduced glutathione; GPx, glutathione peroxidase; HbA1c, glycated hemoglobin; HOMA-B, Homeostatic Model Assessment of β-cell function; HOMA-IR, Homeostatic Model Assessment of Insulin Resistance; hs-CRP, high-sensitivity C-reactive protein; IDO, indoleamine 2,3-dioxygenase; IL, interleukin; iNOS, inducible nitric oxide synthase; i-Tregs, induced regulatory T cells; LGG, *Lacticaseibacillus rhamnosus* GG; LPS, lipopolysaccharide; MDA, malondialdehyde; MIP-1β, macrophage inflammatory protein 1 beta; MMP-9, matrix metalloproteinase 9; MyD88, myeloid differentiation primary response protein 88; NADPH, reduced nicotinamide adenine dinucleotide phosphate; NF-κB, Nuclear factor kappa-light-chain-enhancer of activated B cells; NK, natural killer; NO, nitric oxide; Nrf2, nuclear factor erythroid 2-related factor 2; PDA, polydopamine; RA, rheumatoid arthritis; RANTES, regulated upon activation, normal T-cell expressed and secreted; RCT, randomized controlled trial; ROS, reactive oxygen species; SOD, superoxide dismutase; STZ, streptozotocin; T1D, type 1 diabetes; TAC, total antioxidant capacity; TBG, total blood glucose; Th1, T helper 1 cell; Th17, T helper 17 cell; TLR, Toll-like receptor; TNF-α, tumor necrosis factor alpha; Tregs, regulatory T cells; VAS, Visual Analog Scale; and WBC, white blood cell count.

## 6. Current Challenges, Limitations, and Future Perspectives

The interpretation of probiotic-mediated immunoredox effects is limited by substantial heterogeneity in strains, formulations, doses, treatment duration, and experimental models. Even strains within the same species may differ in metabolic activity, surface properties, metabolite production, and interactions with host immune pathways, limiting extrapolation across preparations. Differences in disease models, disease stages, host characteristics, and the use of single- versus multi-strain formulations further hinder cross-study comparisons. Moreover, many preclinical studies assess antioxidant enzymes, inflammatory mediators, or oxidative damage as isolated endpoints, without establishing temporal or mechanistic links between GM modulation, redox signaling, and disease outcomes. This is particularly relevant to the MMA framework, as changes in individual components are often interpreted as pathway modulation without demonstrating that microbiota alterations precede changes in metabolite profiles that subsequently influence mitochondrial or immune phenotypes.

Clinical translation remains particularly constrained by the limited number of adequately powered randomized trials and by the predominance of systemic surrogate biomarkers. Evidence from human studies is also sparse. Most available clinical studies are small and heterogeneous in probiotic formulation and treatment duration, and few are designed to test the mechanistic sequence proposed within the MMA framework. Thus, clinical findings cannot yet establish whether probiotic-induced immunoredox effects are reproducible in humans or whether they translate into disease-relevant tissue protection. Although reductions in MDA levels and changes in antioxidant defenses, cytokine levels, or other redox-related markers have been reported in some conditions, results are inconsistent, and direct evidence of disease-relevant tissue protection remains scarce. Clinical studies also rarely characterize baseline GM composition, treatment-induced microbial changes, or the abundance of specific metabolites alongside immunoredox outcomes. This limits the ability to determine whether a given probiotic exerts a reproducible microbiota-dependent effect or whether observed changes reflect indirect consequences of reduced inflammation or other host responses.

Across the disorders reviewed, evidence for antioxidant and immune modulation is substantially stronger than evidence directly linking these responses to mitochondrial function. Few studies simultaneously assess GM composition, microbiota-derived metabolites, mitochondrial respiration or membrane potential, ROS production, antioxidant defenses, and immune phenotypes within the same experimental framework. Consequently, proposed connections between probiotics, microbial metabolites, mitochondrial redox homeostasis, and immune regulation remain largely mechanistic hypotheses rather than experimentally established pathways. The critical gap is therefore not a lack of evidence for antioxidant or anti-inflammatory activity, but the absence of integrated experiments demonstrating causality across the MMA.

Safety is another important consideration in the clinical translation of probiotic therapies. Although most clinical trials report only mild-to-moderate, self-limiting adverse events [145,146], live microorganisms pose serious risks in vulnerable populations, including critically ill patients in intensive care units, premature infants, and immunocompromised individuals, in whom the risk of bacterial translocation, bacteremia, or systemic infection may outweigh potential benefits [147,148]. A promising strategy to circumvent these limitations is the use of non-viable microorganisms and their functional metabolites, characterized as postbiotics, which offer targeted immunomodulatory capacity and superior safety profiles [149,150]. Ultimately, moving beyond the empirical use of biotics will require precision microbiome therapies with strain and metabolite formulations tailored to individual redox, immune, and mitochondrial signatures.

Future work should focus on resolving the causal links that remain untested. Longitudinal studies combining strain-resolved microbiota profiling with targeted measurements of microbial metabolites, mitochondrial respiration and membrane potential, and mtROS production, alongside assessments of redox pathways and immune phenotypes could establish the temporal sequence of events following probiotic administration. Causality could then be examined through targeted metabolite supplementation or inhibition, microbial depletion or transfer, and perturbation of mitochondrial pathways, rather than inferred from parallel changes in biomarkers. In clinical studies, adequately powered randomized trials should use standardized probiotic formulations and incorporate these mechanistic measurements alongside predefined disease-relevant outcomes. Attention should be given to mitochondrial respiration, mtROS production, Nrf2-related pathways, glutathione metabolism, and PGC-1α-dependent mitochondrial biogenesis, together with disease-specific functional outcomes. These approaches would clarify whether the sequence linking GM modulation, microbial metabolites, mitochondrial function, and immune regulation within the MMA framework is experimentally reproducible and clinically relevant. This would move the evidence beyond associations between probiotic use and isolated immunoredox biomarkers.

## 7. Conclusions

Probiotics represent a promising approach for immunoredox modulation across disorders associated with oxidative stress and autoimmunity, with preclinical and clinical evidence supporting effects on oxidative damage, antioxidant defenses, inflammatory signaling, and disease-related outcomes. Benefits are particularly evident for selected Lactobacillus and Bifidobacterium strains, although the magnitude and consistency of responses vary substantially according to strain, disease context, formulation, and host characteristics. The evidence reviewed also supports an important role for microbiota-derived metabolites, intestinal barrier function, and immune–redox signaling in shaping probiotic responses, providing a biological basis for considering the microbiota–mitochondria axis (MMA) as a relevant mechanistic framework.

Direct evidence, however, remains insufficient to establish the MMA as the mechanism responsible for probiotic-mediated immunoredox effects. Most studies evaluate microbiota composition, oxidative stress, immune responses, or mitochondrial parameters separately, with few integrating these levels within the same experimental design and even fewer addressing mitochondrial outcomes in clinical populations. Future research should therefore combine longitudinal microbiota and metabolite profiling with assessments of intestinal barrier integrity, mitochondrial bioenergetics, redox signaling, and disease-specific immune and functional outcomes. Establishing this mechanistic connection will be essential to determine whether probiotic modulation of the MMA represents a reproducible therapeutic pathway rather than a plausible association, and to support the development of more precise, strain-specific microbiota-targeted interventions.

## Data Availability

The raw data supporting the conclusions of this article will be made available by the authors on request.

## Abbreviations

8-OHdG	8-Hydroxy-2′-deoxyguanosine
APC	Antigen-Presenting Cell
ATP	Adenosine triphosphate
CAT	Catalase
CFU	Colony-Forming Unit
CNS	Central nervous system
CREM	cAMP-responsive element modulator
CRP	C-reactive protein
CX3CR1	C-X3-C motif chemokine receptor 1
DC	Dendritic Cell
DNA	Deoxyribonucleic acid
eNOS	Endothelial nitric oxide synthase
GM	Gut microbiota
GPx	Glutathione peroxidase
GPx4	Glutathione peroxidase 4
GSDMD	Gasdermin D
GSH	Reduced glutathione
H_2_O_2_	Hydrogen peroxide
HIF-1α	Hypoxia-inducible factor 1-alpha
hs-CRP	High-sensitivity C-reactive protein
IFN-γ	Interferon gamma
IgA	Immunoglobulin A
IL	Interleukin
IL-2	Interleukin 2
IL-6	Interleukin 6
IL-8	Interleukin 8
IL-10	Interleukin 10
IL-18	Interleukin 18
IL-1β	Interleukin 1 beta
iNOS	Inducible nitric oxide synthase
JAK/STAT	Janus kinase/signal transducer and activator of transcription
KEAP1	Kelch-like ECH-associated protein 1
LPS	Lipopolysaccharide
MAPK	Mitogen-activated protein kinase
MDA	Malondialdehyde
MMA	Microbiota–mitochondria axis
MMP-9	Matrix metalloproteinase 9
MPO	Myeloperoxidase
MS	Multiple sclerosis
mtDNA	Mitochondrial DNA
mTOR	Mechanistic target of rapamycin
mtROS	Mitochondrial reactive oxygen species
MyD88	Myeloid differentiation primary response protein 88
NADPH	Nicotinamide adenine dinucleotide phosphate, reduced form
NET	Neutrophil extracellular trap
NETosis	Neutrophil extracellular trap formation
NFAT	Nuclear factor of activated T cells
NF-κB	Nuclear factor kappa-light-chain-enhancer of activated B cells
N-GSDMD	N-terminal fragment of Gasdermin D
NK	Natural Killer
NLRP3	Nucleotide-binding oligomerization domain-, leucine-rich repeat-, and pyrin domain-containing protein 3
NO	Nitric oxide
NOX	NADPH oxidase
NOX2	NADPH oxidase 2
NP	Neuropathic pain
Nrf2	Nuclear factor erythroid 2-related factor 2
ox-mtDNA	Oxidized mitochondrial DNA
PGC-1α	peroxisome proliferator-activated receptor gamma coactivator 1-alpha
PI3K	Phosphoinositide 3-kinase
PI3K-AKT	Phosphoinositide 3-kinase-protein kinase B
PKC	Protein kinase C
PRR	Pattern Recognition Receptors
RNS	Reactive nitrogen species
ROS	Reactive oxygen species
SCFA	Short-chain fatty acid
SLE	Systemic lupus erythematosus
SOD	Superoxide dismutase
TAC	Total antioxidant capacity
TCA	Tricarboxylic Acid
Th1	T helper 1 cell
Th17	T helper 17 cell
TLR	Toll-like receptor
TLR2	Toll-like receptor 2
TLR4	Toll-like receptor 4
TLR7	Toll-like receptor 7
TNF-α	Tumor necrosis factor alpha
Treg	Regulatory T cell
ZO-2	Zonula occludens 2
